# Laos case study

**DOI:** 10.1186/1477-7517-9-29

**Published:** 2012-07-09

**Authors:** Brigitte Tenni, Vanphanom Sychareun

**Affiliations:** 1 Nossal Institute for Global Health, University of Melbourne, Melbourne, Australia; 2 Faculty of Postgraduate Studies, University of Health Sciences, Melbourne, Australia

## Abstract

Peuan Mit is a Lao organization working to address the needs of children and youth living and working on the streets. This case study outlines how a trusted and strong relationship with local police provides mutual benefit.

## Peuan Mit

Peuan Mit (‘friends’ in the Lao language) is a program designed and implemented by Friends International, in partnership with the Ministry of Labor and Social Welfare, to address the needs of street children and youth in Laos. The Peuan Mit project started in May 2004 with the support of the Vientiane Municipality and is the only program of its kind in Lao PDR that responds to the needs of children living and working on the streets [1].

Many of the children with whom Peuan Mit work face very challenging issues such as conflict with parents or the police, drug use, disability, trauma and sex work.

In 2007, Peuan Mit carried out a knowledge, attitude and practice (KAP) survey. Although not a representative sample, it provided useful information about ‘at risk’ and street based children in Vientiane. More than a third (38.7%) of those interviewed reported a history of drug use, with most recent use ranging from over 10 years to 2 weeks. Drugs reportedly used included methamphetamine, opium, heroin, cannabis, solvents (glue), and alcohol. Of those who reported drug use, methamphetamine was most commonly used; all who reported methamphetamine use said that their most recent drug use was smoking methamphetamine [2].

Every month Peuan Mit works with 800 children and youth in Vientiane to prevent homelessness amongst children and help young people reintegrate into Lao society. They support children and young people to return to school, find employment, return to their families, become citizens, to experience culture and to express themselves. A team of 50 Lao staff at Peuan Mit provides a range of services including a mobile school, remedial classes, hygiene facilities, recreational workshops (art, dance, drama, sport) emergency shelter, life-skills education, counselling sessions, vocational training and job placement and family reintegration [3].

Peuan Mit has no formal MoU with the Ministry of Public Security or with operational police, and no regular meetings; however they have developed informal relationships with local police in the course of their work. Peuan Mit invite police to join the twice yearly Project Advisory Committee meeting which includes local counter-parts, Ministries and donors. Peuan Mit works closely with young people in conflict with the law and visits young people incarcerated in drug detention centres and prisons to advocate on their behalf and attempt to link them into Peuan Mit services.

"If they commit a crime and then we meet with them- we make a plan- if they want to come with us then we make a letter to the police station- takes about one month"

Police who know of Peuan Mit’s work refer young people to them. If the young person is under 15 years and the crime committed has no victim, the police may choose to release the young person into their care.

"Now the good thing is that they know us more, they know our staff and it’s good for them to refer to us"

Peuan Mit view relationships with police as an opportunity to facilitate access to young people in need. Without a good relationship with law enforcement gaining access to these vulnerable young people would be all the more difficult.

"“they open the door for us to these people [and] open the discussion”"

Recently a police officer even assisted Peuan Mit to find work for a young person who had been released from detention.

Ultimately Peuan Mit would like to see greater opportunities for diversion for young people and alternatives to incarceration. They are also interested in pursuing an MOU with the Ministry of Public Security to facilitate relations with police.

Police interviewed saw Peuan Mit as providing important services for homeless street children such as life skills and vocational training. They saw Peuan Mit as an important referral option for young people and an alternative to detaining young people with adults especially when clearing the streets before a public event.

Police reported sending children to Peuan Mit if they had no family or if their family could not control their behaviour. They also sent children under 15 years who had been arrested to Peuan Mit which they perceived to be a better outcome than incarceration. Children under 15 years cannot be charged with a crime in Lao PDR.

The Ministry of Labour and Social Welfare represents Peuan Mit within the government and liaises with the Ministry of Public Security where relevant. As the police explain:

"We don’t have a ‘partnership’ with Peuan Mit, we refer to the Ministry of Social Welfare…we work through the Ministry and district police station."

Staff at the Ministry of Labour and Social Welfare stated that children under 18 who have been arrested may be sent by police to Peuan Mit to advocate for them on a case by case basis.

The relationship between police and Peuan Mit is not formalised and is largely based on personal relationships and trust. This has been aided by the reputation that Peuan Mit has developed over time in responding to youth at risk. It demonstrates the additional value of developing positive relationships with law enforcement to achieve better outcomes for young people in conflict with the law including young people who use drugs.

## Competing interests

The authors declare that they have no competing interests.

## Authors’ contributions

BT and VS collected, collated and analysed data and drafted the manuscript. Both authors read and approved the final manuscript.
